# Comparison of the aperture and midportion femoral tunnel widening after anterior cruciate ligament reconstruction: A systematic review and meta-analyses

**DOI:** 10.1097/MD.0000000000016121

**Published:** 2019-06-28

**Authors:** Haluk Celik, Dae-Hee Lee

**Affiliations:** a Department of Orthopaedic Surgery, Umraniye Training and Research Hospital, Istanbul, Turkey; b Department of Orthopaedic Surgery, Samsung Medical Center, Sungkyunkwan University School of Medicine, Seoul, Republic of Korea.

**Keywords:** anterior cruciate ligament reconstruction, aperture, midportion, tunnel widening

## Abstract

**Background:**

To identify whether the aperture or the midportion of the femoral tunnel had a larger tunnel widening in patients who undergo ACL reconstruction.

**Methods:**

PubMed, EMBASE and Cochrane Library were searched for relative studies that evaluated tunnel widening in patients underwent arthroscopic ACL reconstruction. Two reviewers independently recorded data from each study, including the sample size and magnitude of tunnel widening. Random-effects meta-analyses were performed to pool the outcomes of tunnel widening by estimating the standardized mean differences in tunnel widening and their 95% confidence intervals (CIs), Publication bias was assessed using funnel plots and Egger test when the number of included studies was >10.

**Results:**

Eleven included studies compared tunnel widening between the aperture and the midportion. In these studies, 372 and 92 patients underwent single- and double-bundle ACL reconstructions, respectively. Subgroup analyses in terms of evaluation imaging tool for tunnel widening showed no significant differences in tunnel widening between the aperture and the midportion of the femoral tunnel regardless of plain radiograph (mean difference, 0.02 mm; *P* = .97), computed tomography (mean difference 0.08 mm; *P* = .55), and magnetic resonance imaging (mean difference, 0.13 mm; *P* = .78). Likewise, no significant difference in femoral tunnel widening was found between the aperture and the midportion, irrespective of transtibial (mean difference, 0.18 mm; *P* = .57), anteromedial (mean difference, 0.02 mm; *P* = .90), and outside-in techniques (mean difference, 0.01 mm; *P* = .98).

**Conclusion:**

No significant difference in femoral tunnel widening was found between the aperture and the midportion in the patients who underwent ACL reconstruction.

## 1 Introduction

Femoral tunnel widening is a well-known phenomenon in patients with anterior cruciate ligament (ACL) reconstruction. Although femoral tunnel widening does not seem to affect clinical outcomes in a short period,^[1,2]^ many orthopedic surgeons want to reduce its occurrence because when severe, it can complicate the revision surgery.^[3–6]^ Although tunnel widening may be caused by a combination of biological and biomechanical factors,^[5]^ graft-tunnel interface micromotion, which is the main biomechanical factor, was found to be the predominant theoretical reason for tunnel widening in a previous literature.^[7]^ This graft motion within the femoral tunnel could abrade the edge of the tunnel aperture and lead to a wider tunnel enlargement in the aperture than in the midportion of the femoral tunnel.^[3]^ Therefore, whether tunnel widening really occur more widely in the aperture than in the midportion of the femoral tunnel should be determined to produce evidence on the role of the biomechanical factor in the occurrence of tunnel widening after ACL reconstruction. However, previous literatures showed conflicting results for which between the aperture and the midportion of the femoral tunnel has a larger tunnel widening. In addition, standardization of the fixation material and measurement imaging tool for tunnel widening is lacking. Therefore, the purpose of this study was to identify which between the aperture and the midportion of the femoral tunnel had a larger tunnel widening in patients who underwent ACL reconstruction. We hypothesized that the aperture portion would have a larger tunnel widening than the midportion of the femoral tunnel.

## 2 Materials and methods

### 2.1 Literature search

The study design was based on the Cochrane Review Methods. In accordance with the guidelines of the PRISMA (Preferred Reporting Items for Systematic Reviews and Meta-analyses) statement, multiple comprehensive literature databases, including PubMed, Embase, and Cochrane Library, were searched for studies that evaluated tunnel widening in patients who underwent arthroscopic ACL reconstruction. No restrictions on language or year of publication were established. The search terms used in the title, abstract, MeSH, and keywords fields included (ACL OR anterior cruciate ligament reconstruction) AND (ACL OR tunnel widening) AND (anterior cruciate ligament reconstruction OR tunnel enlargement).The latest search was done on July 2018.

### 2.2 Study selection

Two reviewers evaluated the titles and abstracts of the retrieved papers and selected relevant studies for a full review. If the abstract did not provide sufficient data to decide, the complete article was reviewed. Studies were included in the analysis if they

1. included patients who underwent primary arthroscopic ACL reconstruction;
2. evaluated the femoral tunnel widening with validated imaging tools such as plain radiography, computerized tomography, and magnetic resonance imaging;
3. completed reported parameters, including means, standard deviations, and sample numbers.

In assessing and organizing pooled studies, the country and city of the hospital, or institution, at which the arthroscopic surgeries were performed and the operating surgeon's name in the studies and the evaluation period were checked to exclude duplicate cohorts of patients. If the same patient cohort was evaluated in more than one study, the latest study with the longest follow-up period was included, whereas the others were excluded. Institutional review board approval and patient informed consent (written/oral) were not required because all analyses were based on previously published studies.

### 2.3 Data extraction

Two investigators independently extracted data from each study using a predefined data extraction form. Any disagreements unresolved by discussion were reviewed by a third investigator, if needed. The main outcome of interest included the size of femoral tunnel widening, which was calculated as a change in femoral tunnel diameter based on immediate postoperative imaging. The number of femoral tunnels (single or double bundle), the surgical technique (transtibial, anteromedial portal, and outside-in), and basic demographic data, including age, sex, and time interval from surgery to measurement of tunnel widening were also recorded for each included study.

### 2.4 Assessment of methodological quality

The subsections that compose the Coleman methodology score are based on the subsections of the CONSORT (Consolidated Standards of Reporting Trials) statement for randomized controlled trials but were modified to allow for other study designs. The original Coleman methodology score, which was developed for surgical treatment of tendinopathy, was modified for arthroplasty of the knee. The quality of each included study was evaluated by 2 independent investigators using the modified Coleman methodology score (MCMS).

### 2.5 Statistical analysis

The main outcomes of the meta-analysis were the mean differences in tunnel widening after ACL reconstruction between the aperture and the midportion of the femoral tunnel. Tunnel widening was measured using an imaging modality such as plain radiography, computed tomography (CT), or magnetic resonance imaging (MRI), and was defined by the change in tunnel enlargement as compared with the immediate postoperative status. Random-effects meta-analyses were performed to pool the outcomes of tunnel widening across the included studies by estimating the standardized mean differences in tunnel widening and their 95% confidence intervals (CIs) between the aperture and the midportion of the femoral tunnel. Heterogeneity was determined by estimating the proportion of between-study inconsistencies due to actual differences between studies, rather than differences due to random error or chance, using the *I*^2^ statistics, with values of 25%, 50%, and 75% considered low, moderate, and high, respectively. Publication bias was assessed using funnel plots and Egger test when the number of included studies was >10 for each variable. A meta-regression analysis was performed to assess the effect of the age, sex, and follow-up period on tunnel widening difference in the aperture and the midportion of the femoral tunnel. All statistical analyses were performed using RevMan version 5.2 (Copenhagen, the Nordic Cochrane Centre, The Cochrane Collaboration, 2012).

## 3 Results

### 3.1 Identification of studies, and study characteristics and quality

**Figure 1:**
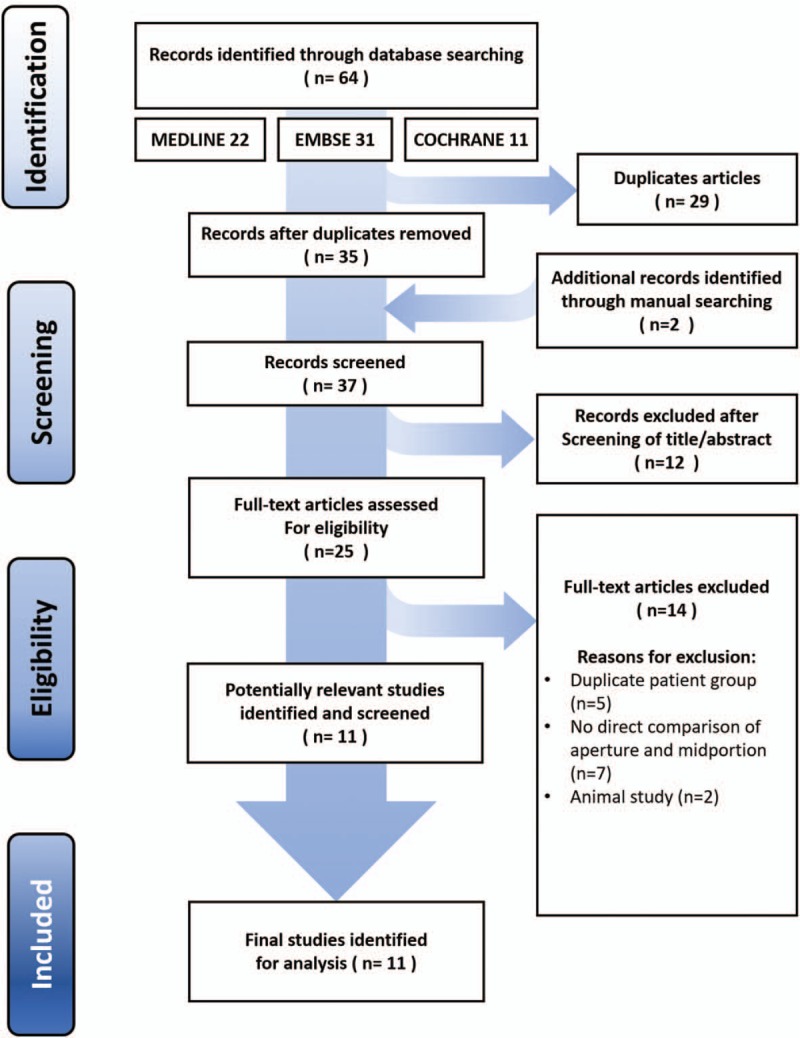
PRISMA (Preferred Reporting Items for Systematic reviews and Meta-analyses) flow diagram of the identification and selection of the studies included in the meta-analysis.

An initial electronic search yielded 35 studies, and an additional two publications were identified through manual searching. After applying our inclusion and exclusion criteria, we included 11 studies in the meta-analysis. Figure 1 shows the details of study identification, inclusion, and exclusion.

**Figure 2:**
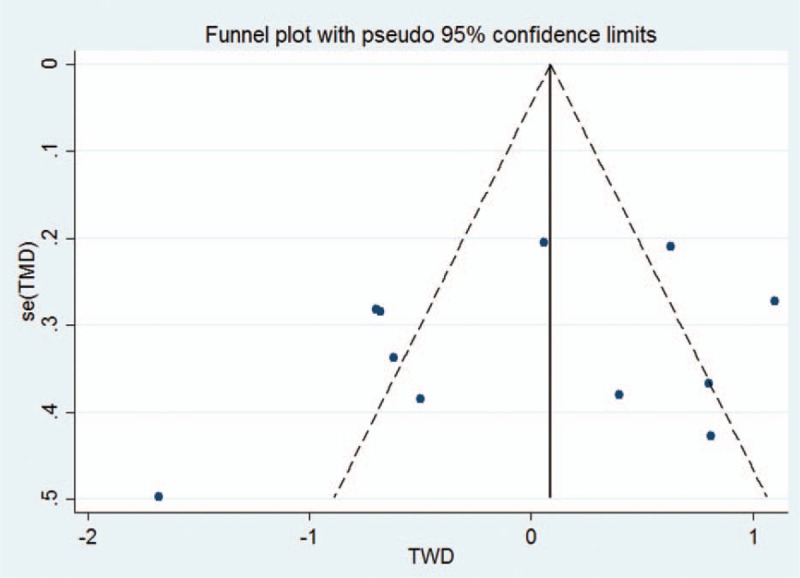
Funnel plots showing relatively symmetrical tunnel widening difference (TWD) between aperture and midportion of femoral tunnel after single bundle ACL reconstruction.

In terms of the measurement tool for tunnel widening, six studies used CT; 3, MRI; and remaining 2, plain radiography. Of the 11 included studies, 10 used the autologous hamstring tendon and only one used the allo-tibialis tendon. Seven studies performed single-bundle ACL reconstruction, two performed double-bundle studies, and two performed both single- and double-bundle ACL reconstructions. Patient characteristics, time interval from immediate postoperative status to tunnel widening measurement, age, body mass index, sex distribution, surgical technique regarding graft choice and femoral drilling technique, and MCMS are summarized in Table 1. The total mean (SD) MCMS of the included studies was 63 (11.5) [range, 44–84] of 100, regarded as fair quality. Of the 11 studies, four had a mean MCMS of >70, while three had a mean score of <55. In general, evaluation of publication bias is unnecessary when <10 studies are included. Therefore, we only assessed the publication bias of studies reporting tunnel widening difference between aperture and midportion after single bundle ACL reconstruction, with funnel plots showing that the tunnel widening difference between 2 locations were relatively symmetric (Fig. 2), indicating lack of publication biases among the included studies. Egger test also revealed no significant publication bias in tunnel widening difference (*P* = .379).

**Table 1:**
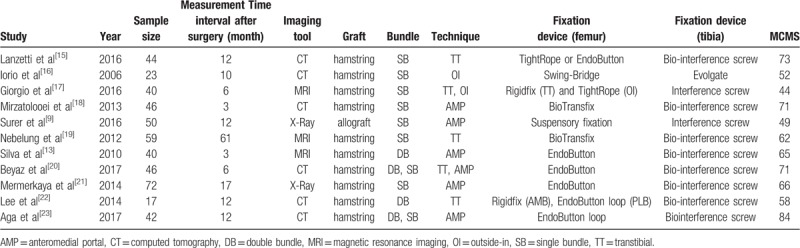
Characteristics of the included studies.

### 3.2 Comparison of tunnel widening between the aperture and the midportion

**Figure 3:**
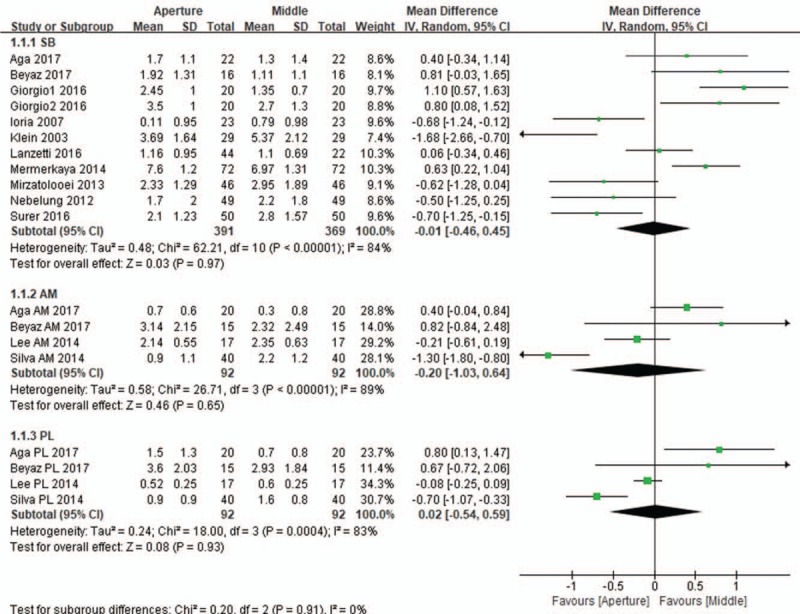
Forest plot showing the mean differences in tunnel widening between the aperture and the midportion of the femoral tunnel in single- and double-bundle ACL reconstructions. The subgroup analysis results of single-bundle reconstruction showed that the difference in tunnel widening between the aperture and the midportion was not significant (mean difference, 0.01 mm; *P* = .97). No significant differences in tunnel widening were found in both the anteromedial tunnel (mean difference, 0.20 mm; *P* = .65) and posterolateral tunnel (mean difference, 0.02 mm; *P* = .93) in double-bundle reconstruction.

**Figure 4:**
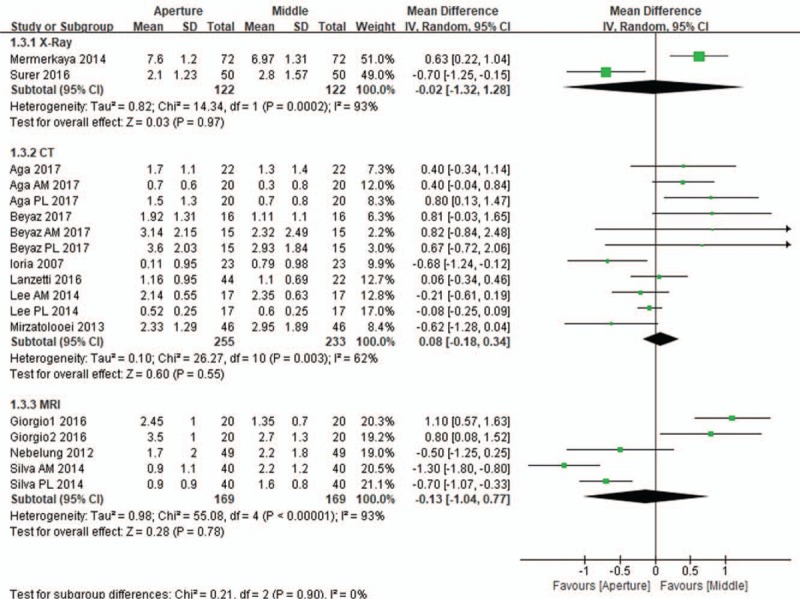
Forest plot showing the mean differences in tunnel widening between the aperture and the midportion of the femoral tunnel according to imaging tool. No significant differences in tunnel widening were observed between the aperture and the midportion of the femoral tunnel regardless of whether plain radiography (mean difference, 0.02 mm; *P* = .97), CT (mean difference, 0.08 mm; *P* = .55), or MRI (mean difference, 0.13 mm; *P* = .78) was used.

**Figure 5:**
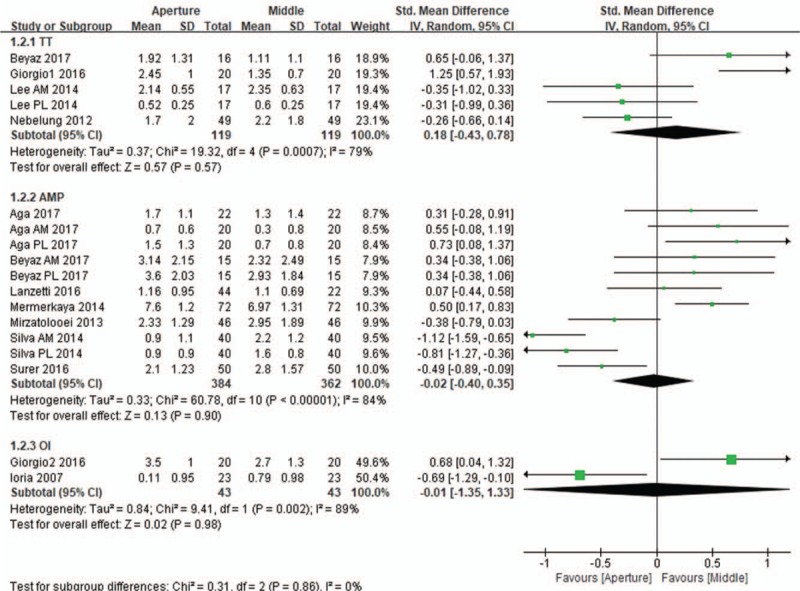
Forest plot showing the mean differences in tunnel widening between the aperture and the midportion of the femoral tunnel according to the surgical technique used in ACL reconstruction. No significant difference in femoral tunnel widening was found between the aperture and the midportion irrespective of the transtibial (mean difference, 0.18 mm; *P* = .57), anteromedial (mean difference, 0.02 mm; *P* = .90), and outside-in techniques (mean difference, 0.01 mm; *P* = .98).

All 11 included studies compared tunnel widening between the aperture and the midportion. In these studies, 372 and 92 patients underwent single- and double-bundle ACL reconstructions, respectively. The subgroup analysis of the subjects who underwent single-bundle reconstruction revealed that the difference in the mean change in tunnel widening from the immediate postoperative status to the last follow-up between the aperture and the midportion was not significant (mean difference, 0.01 mm; 95% CI, −0.46 to 0.45; *P* = .97; *I*^2^ = 84%). Similar findings were found in the pooled mean changes in tunnel widening in the subgroup of patients who received double-bundle reconstruction. No significant differences in tunnel widening were found in both the anteromedial tunnel (mean difference, 0.20 mm; 95% CI, −1.03 to 0.64; *P* = .65; *I*^2^ = 89%) and posterolateral tunnel (mean difference, 0.02 mm; 95% CI, −0.54 to 0.59; *P* = .93; *I*^2^ = 83%; Fig. 3). The subgroup analyses of the evaluation imaging tools used for tunnel widening also revealed no significant differences in tunnel widening between the aperture and the midportion of the femoral tunnel, regardless of whether plain radiography (mean difference, 0.02 mm; 95% CI, −1.32 to 1.28; *P* = .97; *I*^2^ = 93%), CT (mean difference, 0.08 mm; 95% CI, −0.18 to 0.34; *P* = .55; *I*^2^ = 62%), or MRI (mean difference, 0.13 mm; 95% CI, −1.04 to 0.77; *P* = .78; *I*^2^ = 93%; Fig. 4) was used. Likewise, the subgroup analysis of the surgical technique also showed similar femoral tunnel widening between the aperture and the midportion, irrespective of the transtibial (mean difference, 0.18 mm; 95% CI, −0.43 to 0.78; *P* = .57; *I*^2^ = 79%), anteromedial (mean difference, 0.02 mm; 95% CI, −0.40 to 0.35; *P* = .90; *I*^2^ = 84%), and outside-in techniques (mean difference, 0.01 mm; 95% CI, −1.35 to 1.33; *P* = .98; *I*^2^ = 89%; Fig. 5).

### 3.3 Meta-regression analyses

The results of the meta-regression analyses are reported in Table 2. Demographic data, including patient sex and age, and length of follow-up period, were not significantly associated with the difference in tunnel widening between the aperture and the midportion of the femoral tunnel after ACL reconstruction.

**Table 2:**
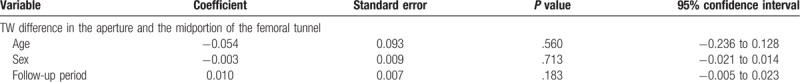
Meta-regression analysis for comparison of the associations among age, sex, and follow-up periods with the differences in tunnel widening between the aperture and the midportion of the femoral tunnel in patients who underwent anterior cruciate ligament reconstruction.

## 4 Discussion

The most important finding of this study was that femoral tunnel widening showed no significant difference between the aperture and the midportion, and these trends were not affected by the surgical techniques and measurement imaging tools used.

Femoral tunnel widening is a bone resorption phenomenon that occurs around the tunnel created by a drill reamer. Although the exact causes of femoral tunnel widening after ACL reconstruction were not identified because of multifactorial affecting factors, most literatures suggested 2 plausible reasons that could be divided into biomechanical and biological factors. Both factors favor greater tunnel widening in the aperture than in the midportion of the femoral tunnel. Biomechanical factors, represented by graft motion in the tunnel, were traditionally more weighted than biological factors as a predominant etiology of tunnel widening. Graft micromotion in the tunnel could occur longitudinally (bungee effect) and transversely (windshield-wiper effect) when referring to the longitudinal axis of the tunnel. Several biomechanical studies demonstrated that the longer the distance from the articular surface to the graft fixation, the greater the transverse graft micromotion at the tunnel aperture and the longitudinal graft micromotion in the tunnel cylinder. The transverse motion of the tunnel aperture could abrade the edge of the tunnel aperture, resulting in greater widening in the aperture than the midportion of the tunnel.^[8]^ The greater aperture widening was supported not only by a biomechanical study, but also by a clinical study. In a clinical study of 50 patients who underwent single-bundle ACL reconstruction with suspensory femoral fixation, Surer et al^[9]^ showed that the tunnel widening was 0.3 to 10 mm greater in the aperture than in the midportion of the femoral tunnel regardless of using a fibrin clot. The main theory of the mechanism of the biological factor of tunnel widening was local inflammation to the localized cell necrosis of the ACL graft over time after surgery. This change in the biological environment by localized inflammation due to graft necrosis could release cytokines, thus triggering a resorptive effect of osteoclast on the bone adjacent to the tunnel. Rodeo et al^[10]^ performed ACL reconstruction in 15 rabbits and counted the number of osteoclasts in the aperture and midportion of the tunnel on serial sections. Their result demonstrated that osteoclasts were more numerous at the tunnel aperture than at the midportion. Given the biomechanical and biological causes of tunnel widening in theory and the supporting evidence in the literature, the size or magnitude of tunnel widening after ACL reconstruction would be more likely greater in the aperture than in the midportion of the tunnel. However, contrary to our hypothesis, the results of the present study did not indicate a significant difference in tunnel widening between the aperture and the midportion of the tunnel.

Although the reason for the insignificant difference in tunnel widening between the aperture and the midportion of the tunnel is unclear, several possible reasons could be named. One possible reason assumes that a biological reaction occurs in the whole interface between the graft and the bone tunnel by synovial fluid invasion to the graft-tunnel interface (synovial fluid bathing), instead of being limited to the aperture of the tunnel. As described earlier, Rodeo et al^[10]^ reported that osteoclasts were more predominant in the aperture than in the midportion, but the time point of measuring the number of osteoclasts, which is 1 week after operation, was too short, even when considering the animal study. The mean period of tunnel widening after ACL reconstruction in the human clinical study was 3 months after operation. Over the postoperative course, graft necrosis could widen the graft/tunnel interface more than the immediate postoperative status. This situation could lead to easy access of joint synovial fluid, which contains osteolytic cytokine, into the graft-tunnel interface from the aperture to the midportion and end of the tunnel.^[11,12]^ Another potential cause could be the difference in bone density between the aperture and the midportion. The cancellous bone of the central tunnel is softer than the denser bone of the cortex, and the midportion of the tunnel may be more vulnerable to bone abrasion or erosion by graft motion or local inflammation than the aperture.^[13]^ A difference or change of mechanism in the biomechanical factor also could be a possible explanatory cause of the lack of a significant difference in tunnel widening between the aperture and the midportion. A recent clinical imaging study^[14]^ classified the pattern of tunnel widening as linear, cavity, mushroom, and cone types. The study showed that among the 4 different types, the linear shape was the most common pattern of tunnel widening. This finding indicated that the longitudinal graft motion (bungee effect) might have a greater contribution to tunnel widening than transverse graft motion (windshield wiper effect).

### 4.1 Limitations

This study had several limitations. Tunnel widening was quantified in this study as the change in tunnel size from the immediate postoperative period to a certain time point after surgery. In case of absent information for immediate postoperative tunnel size, the drill reamer size was considered as the immediate postoperative tunnel size. This substitution may result in error in the accuracy of the immediate postoperative tunnel size. Another limitation was that we could not entirely exclude other factors that may have affected tunnel widening, such as graft type, tunnel widening measurement time after surgery, and tibial fixation type. However, we tried to reduce heterogeneity by only including the suspensory mechanism of the femoral fixative (cross pin or extracortical suspension apparatus) and soft tissue graft (auto-hamstring or allo-tibialis tendons). In addition, the results of the meta-regression analysis in our study showed no correlation between the differences in tunnel widening between the aperture and the midportion and the time point of tunnel widening measurement after surgery.

### 4.2 Future directions

Despite these limitations, the results of the present study may provide important baseline data on tunnel widening for further studies assessing any possible correlation between femoral tunnel widening and clinical outcomes or subsequent residual laxity after ACL reconstruction. Our study also could alleviate the potential concerns associated with femoral tunnels being wider in either midportion or aperture.

## 5 Conclusions

In conclusion, no significant difference in femoral tunnel widening was found between the aperture and the midportion in the patients who underwent ACL reconstruction.

## Author contributions

**Conceptualization:** Dae-Hee Lee.

**Data curation:** Haluk Celik.

**Formal analysis:** Dae-Hee Lee.

**Investigation:** Haluk Celik.

**Methodology:** Dae-Hee Lee.

**Software:** Dae-Hee Lee.

**Writing – original draft:** Haluk Celik, Dae-Hee Lee.
